# Effect of Local Warm Compression on Restless Leg Syndrome and Fatigue among Critical Care Nurses: A Parallel Randomized Clinical Trial

**DOI:** 10.1155/2022/7330308

**Published:** 2022-08-27

**Authors:** Maryam Ameri, Hossein Ebrahimi, Ahmad Khosravi, Seyedmohammad Mirhosseini, Mohammad Reza Khatibi

**Affiliations:** ^1^Student Research Committee, School of Nursing & Midwifery, Shahroud University of Medical Sciences, Shahroud, Iran; ^2^Department of Nursing, Center for Health Related Social and Behavioral Sciences Research, Shahroud University of Medical Sciences, Shahroud, Iran; ^3^Department of Epidemiology, Center for Health Related Social and Behavioral Sciences Research, Shahroud University of Medical Sciences, Shahroud, Iran; ^4^School of Nursing and Midwifery, Shahroud University of Medical Sciences, Shahroud, Iran

## Abstract

**Methods and Materials:**

This parallel randomized clinical trial was conducted on 120 CCNs in Shahroud by the census sampling method. Inclusion criteria included suffering from restless leg syndrome and having no wound or inflammation over the organ. The participants were assigned into two groups by the use of quadruple blocks. The intervention group received the warm compress for 12 sessions lasting 4 weeks and the control group did not receive an intervention. Data were collected using multidimensional fatigue inventory (MFI) and the Restless Legs Syndrome Scale and then analyzed using descriptive and inferential statistics (chi-squared test, independent sample *t*-test, and pair sample *t*-test).

**Results:**

The two groups were homogeneous in terms of demographic characteristics. Prior to the intervention, the two groups of warm compression and control did not have a significant difference in terms of mean fatigue and restless leg syndrome scores; however, after the intervention, a significant reduction was observed in the intervention group (*p* < 0.001).

**Conclusion:**

According to the results of the current study, the use of warm compression is an effective intervention in alleviating fatigue and restless leg syndrome, so it is recommended to implement this intervention as a nonpharmacological strategy among CCNs. *Clinical Trial Registration Number*. IRCT20190723044316N1.

## 1. Introduction

Critical care nurses (CCNs) are exposed to stress since they provide end-of-life care, complicated life support, postmortem care, and painful procedures for patients. The experience of high stress levels in CCNs may lead to depression, anxiety, burnout syndrome, and in severe cases, posttraumatic stress disorder [1]. The theory of unpleasant symptoms is often used to demonstrate the complex nature of symptom experiences. Based on this theory, variables such as physiological and psychological factors (such as anxiety and depression) are associated with each other and influence the occurrence of symptoms such as fatigue. The effects of physiological factors, such as sleep disorders and poor physical health, have been well proven on fatigue, and good evidence is available that, for example, indicates poor sleep predicts higher levels of fatigue in nurses [2–4]. Fatigue is defined as debilitating periods of exhaustion that interfere with normal activities [5] and is known as a growing concern for nurses, particularly those who care for critically ill patients [6]. As such, the overall prevalence rates of personal-related fatigue and work-related fatigue in nurses are estimated to be 41.4 and 39.1%, respectively [7]. Other common problems in CCNs include restless leg syndrome (RLS) [8, 9] or Willis-Ekbom Disease (WED), which is a chronic, progressive sensory-motor disorder characterized by uncomfortable and sometimes painful sensations with uncontrollable urge to move the legs [10]. RLS is usually diagnosed based on four main criteria, namely, the tendency to move the legs often associated with unpleasant sensation in the legs, induction or exacerbation of symptoms with rest, relief of symptoms in activity, and daily fluctuations in symptoms with exacerbation in the evening and nighttime hours [11]. RLS is associated with various factors, such as anxiety, depression, sleep quality, and fatigue [12, 13].

Fatigue and RLS have been investigated in several studies to apply supportive interventions, including pharmacological and nonpharmacological groups [14, 15]. Given the limitations of current pharmacological interventions, effective nonpharmacological and noninvasive treatments play a crucial role in the treatment of this problem. Several nonpharmacological treatments, including massage, yoga, and cognitive-behavioral interventions, have been recommended for people suffering from RLS and fatigue. In addition, heat therapy (thermotherapy) is used to relieve the symptoms of this disorder [14, 16, 17]. A variety of methods, such as local heat therapy, sauna, and spa bath, are used for heat therapy [18]. The use of local heat with a warm compress increases skin temperature and thus raises blood circulation, leading to more nutrition and oxygen supply, which can be effective in the treatment of RLS [19]. A warm compress is also effective in the reduction of fatigue and its negative consequences such as a decrease in the quality of life [20]. Although the heat application mechanism has been based on various methods in previous studies, their overall results generally indicate that heat application effectively and favorably influences the relief of RLS and fatigue in a wide range of people. For example, Jafarimanesh et al. (2020) showed that daily placement of the foot in cold or hot water for 10 min (depending on women's preference) for 2 weeks significantly affected the relief of RLS symptoms in pregnant women [21]. Similarly, Shahpasand et al. (2020) reported that the use of a local warm compress at 50°C on the chest twice a day could effectively reduce fatigue in patients with chronic obstructive pulmonary disease [22].

There are limited studies on the effectiveness of warm compresses on negative consequences such as RLS and fatigue among people with stressful occupations (e.g., CCNs). Besides, necessary support should be provided for these people due to the nature of their profession. Therefore, the present study aimed to determine the effectiveness of local warm compresses in RLS and fatigue of CCNs. So, our research was based on the hypothesis that the local warm compresses will alleviate the fatigue and RLS symptoms among CCNs.

## 2. Materials and Methods

### 2.1. Study Design

The present controlled randomized trial was conducted in parallel with a pre/posttest design in 2019, and was approved with the code IRCT20190723044316N1 in the Iranian clinical trial registry system.

### 2.2. Participants

**Figure 1:**
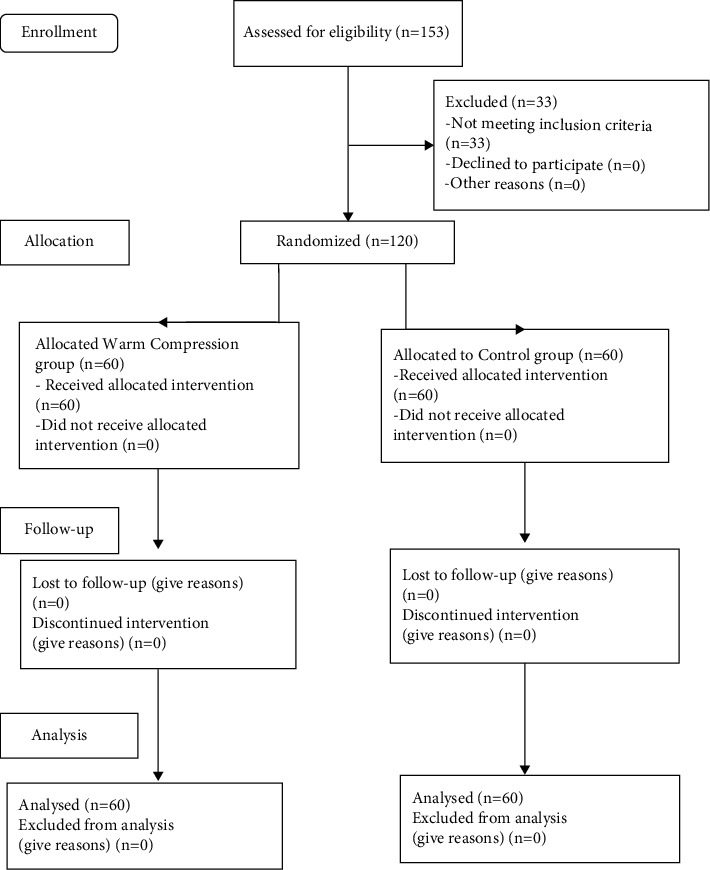
CONSORT flow diagram of the study.

In the present study, the participants consisted of 120 nurses working in the critical care units of hospitals in Shahroud city, northeastern Iran. Nurses were selected sequentially, and those meeting the inclusion criteria were included in the study after completing written consent forms. Eligible participants were divided into two intervention and control groups using quadruple blocks. A trained nursing expert enrolled and identified the participants with RLS (Figure 1). Inclusion criteria were at least 1 year of work experience, holding a bachelor's degree in nursing, suffering from RLS, and working in critical care units. Exclusion criteria were the use of painkillers and narcotics within 72 hours before the study, suffering from psychiatric disorders, neuromuscular disorders, arthritis, vascular disease, diabetes, pregnancy, as well as people with ulcers, sores, and inflammation of the limbs.

### 2.3. Measurements

The required data were collected using a demographic profile form, the Multidimensional Fatigue Inventory (MFI), and the International Standard RLS Scale .

### 2.4. Demographic Characteristics Profile

Demographic characteristics included age, sex, employment status, type of shift, work experience, critical care work experience, overtime hours per month, and having a second job.

### 2.5. RLS Questionnaire

The RLS Questionnaire was first designed by the International RLS Study Group (2003). The standard RLS Questionnaire consists of 10 four-point questions with minimum and maximum scores of 0 and 40, respectively. Scores of 0-10, 11-20, 21-30, and 31-40 indicate mild, moderate, severe, and very severe syndrome, respectively. The reliability of the RLS Questionnaire was evaluated in the International RLS Study Group by the internal consistency method, with a Cronbach's alpha coefficient of 0.93-0.98 [23]. Farajzadeh et al. (2016) assessed the reliability of the Persian version of the RLS screening tool by calculating a Cronbach's alpha of 0.75 [24].

### 2.6. Multidimensional Fatigue Inventory (MFI)

The MFI consists of 20 items, and examines five dimensions of general fatigue, physical fatigue, reduced activity, mental fatigue, and reduced motivation. The questionnaire is scored on a 5-point Likert scale from 1 to 5, and a score from 1 to 5 can be calculated for each item. The lowest and highest obtainable scores are 20 and 100, respectively, and a higher score indicates higher levels of fatigue [25]. The reliability of the Persian version of MFI was confirmed by Cronbach's alpha method. The reliability of the scale was reported to be 0.88 using Cronbach's alpha coefficient by Aghamohammadi and Abazari [26]. Data collection tools were completed by participants in two stages before and after the intervention (at the end of the fourth week).

### 2.7. Intervention

In the present study, local warm compresses were applied to CCNs for 20 min [27] at night hours for 12 sessions in 4 weeks (three sessions a week). Bags filled with warm water (40-43°C) up to one-third to two-thirds of the capacity were used in the intervention group. Warm compresses were applied alternately to the back of both legs of nurses (on the gastrocnemius muscle) so that the bags covered the surface of each leg depending on their body size. As this syndrome occurs at night, warm compresses were used personally by the subjects both in shifts and out of shifts. No intervention was applied to the control group during the study.

### 2.8. Sample Size

According to Nasiriani and Eftekhari [27], a sample size of 120 individuals (60 subjects per group) was determined considering the 95% confidence interval and 80% test power using a formula comparing before and after mean scores while considering the drop of samples.

### 2.9. Blinding

In this study, the data collector and the statistical analyst were blinded to the allocation of individuals to intervention and control groups depending on the type of intervention.

### 2.10. Data Analysis

Data were analyzed using descriptive statistics (absolute and relative frequency, mean, and standard deviation), and inferential statistics (independent *t*-test, chi-squared test, and paired *t*-test). A significance level of *p* < 0.05 was considered for all statistical tests.

### 2.11. Ethical Considerations

This study was approved (ID IR.SHMU.REC.1398.79) by the ethics council of Shahroud University of Medical Sciences. Prior to the study, the implementation method was explained to all participants who were assured about the confidentiality of their information. In addition, they approved the contents of informed consent forms. They were also informed that they were free to withdraw from the study. To observe the ethical considerations, the educational materials for warm compresses were provided to the nurses in the control group in the form of an educational pamphlet after the study.

## 3. Results

The results showed that the mean of the two groups in terms of age were 34.4 ± 6.6 and 34.2 ± 7.2 years in the intervention and control groups, respectively. An independent *t*-test revealed no significant difference between the warm compression and control groups in terms of age and monthly overtime variables. According to the results, the intervention and control groups were almost similar without significant differences in terms of marital status (*p*=0.18), employment status (*p*=0.86), shift type (*p*=0.09), work experience (*p*=0.06), critical care work experience (*p*=0.49), and having a second job as a nurse (*p*=0.57) (Table 1).

The results indicated that the mean scores of RLS were 25.0 ± 5.89 and 24.2 ± 3.6 in the warm compression and control groups, respectively (*p*=0.41). After the intervention, the mean scores of RLS in the intervention (19.2 ± 4.0) and control (23.2 ± 4.3) groups were significantly different (*p* < 0.001) based on the independent *t*-test. The results of paired *t*-test revealed a significant decrease in the score of RLS (*p* < 0.001) after the application of warm compresses in the intervention group, while it was not observed in the control group (Table 2).

According to the results in Table 3, the mean scores of fatigue before the intervention in the warm compression and control groups were 63.4 ± 6.8 and 63.4 ± 6.0, respectively, and the independent *t*-test showed no significant difference between the two groups (*p*=0.97). After the intervention, the mean scores of fatigue in the warm compression (59.6 ± 4.4) and control (63.1 ± 5.6) groups were significantly different between the two groups (*p* < 0.001) based on the independent *t*-test. The results of paired *t*-test showed that the mean fatigue score decreased significantly (*p* < 0.001) after using warm compresses while this decrease was not significant in the control group (*p*=0.78).

## 4. Discussion

The results of this study demonstrated a significant decrease in the mean score of RLS in nurses of the warm compression group after the intervention compared to that before the intervention, but there was no significant difference compared to before the intervention in the control group. In this regard, Nasiriani and Eftekhar reported the application of warm compresses reduced the severity of RLS in hemodialysis patients [27]. Jafarimanesh et al. also found that heat and cold therapy reduced the symptoms of RLS [21]. Park et al. presented evidence that the use of heat therapy and the MMF07 foot massage device could effectively improve RLS symptoms in patients with this disorder [28].

As a strategy in nonpharmacological interventions, heat therapy is used as an effective approach to improving cardiovascular function, relaxation of muscles, improving local blood flow, and increasing joint range of motion [29, 30]. Other nonpharmacological interventions, such as aromatherapy, acupuncture, cognitive-behavioral therapy (CBT), yoga, physical exercise, sleep hygiene training, vibration, and massage techniques, have been used to relieve the symptoms of RLS [14]. For example, Ajorpaz et al. claimed that massage with glycerin oil and lavender oil could effectively reduce RLS in hemodialysis patients [31]. Similarly, Ghasemi et al. showed that the use of aromatherapy foot massage was effective in the reduction of RLS symptoms in women undergoing hemodialysis [32]. Although the mechanism and causes of RLS are different in CCNs and hemodialysis patients, it should be borne in mind that complementary medicine interventions (e.g., local heat therapy or massage without or with aromatherapy) cause a sense of comfort and relaxation in the RLS-affected organ, eventually reducing the symptoms of this syndrome.

The present results demonstrated a significant decrease in the fatigue score after the intervention compared to that of the control group. Similarly, Ozdemir et al. showed that the daily use of a foot bath in warm saltwater for a week could significantly reduce the fatigue caused by chemotherapy [33]. Masuda et al. also reported that the use of heat therapy for 15-25 sessions significantly improved patients with chronic fatigue syndrome so that no recurrence or exacerbation of symptoms was observed within one year after the intervention [34], which is in line with the results of the present study. Rambod et al. also showed that the use of foot reflexology as a nursing intervention significantly affected fatigue and pain relief and improved sleep quality in patients with lymphoma [35]. The findings of Dikmen and Terzioglusuggested the effectiveness of foot reflexology and progressive muscle relaxation on fatigue, pain relief, and the improved quality of life during chemotherapy in patients with gynecologic cancer [36]. The calming effects of warm compresses on RLS and fatigue may result from increased blood supply to the muscles, increased metabolism of body tissues, improved nutrition status, and cell waste disposal. Although these interventions have been conducted on different patients, and their target populations differ from the present study, the findings of these studies support warm compresses and other nonpharmacological treatments on the foot as a complementary therapy to relieve the unpleasant symptoms of RLS and fatigue in CCNs.

Some characteristics of the participants, such as the ratio of nurse to patient, average working hours per week, number of days off per week, and number of night shifts per month, as well as some variables possibly affecting fatigue and RLS (e.g., sleep quality), were not controlled because the working conditions of Iranian nurses are variable every month. Despite these limitations, the findings of the present study are of importance in research and practical applications.

## 5. Conclusions

The use of warm water compresses can effectively reduce the symptoms of RLS and fatigue in CCNs. Therefore, it is recommended to use this easy and inexpensive intervention to alleviate the abovementioned problems.

## Figures and Tables

**Table 1:** Demographic characteristics of CCNs.

Variables		Intervention (*N* = 60)		Control (*N* = 60)		*p* value
		*N*	%	*N*	%	
Marital status	Single	49	87.1	42	71.2	0.18
	Married	11	18.3	17	28.8	

Employment status	Formal employment	29	48.3	32	54.2	0.86
	Contract employment	15	25	15	25.4	
	Human resources plan	10	16.7	8	13.6	
	Contractual employment	6	10	4	6.8	

Shift	Fixed shift	1	1.7	5	8.5	0.09
	Shift rotation	58	98.3	54	91.5	

Work experience (year)	≤5	19	32.2	10	16.9	0.06
	6-10	24	40.7	23	39	
	11-15	5	8.5	16	27.1	
	16-20	8	13.6	8	13.6	
	21-25	3	5.1	2	3.4	

Critical care work experience (year)	≤5	42	70	39	66.1	0.49
	6-10	16	26.7	15	25.4	
	11-15	2	3.3	5	8.5	

Second job	Yes	2	3.3	1	1.7	
	No	58	96.7	58	98.3	

Age (year)		34.4 ± 6.6	34.6 ± 7.2	0.87		
Overtime hours per month (hour)		43.4 ± 24.5	45.8 ± 22.3	0.57		

*N*: frequency; %: percent; *p*: *p* value; CCN: critical care nurse.

**Table 2:** The mean score of RLS before and after intervention in participants of both groups.

Variable		Intervention (*N* = 60)	Control (*N* = 60)	Intergroup test results
		Mean ± SD	Mean ± SD	
RLS	Preintervention	25.0 ± 5.9	24.22 ± 3.6	*p*=0.41^*∗*^
	Postintervention	19.2 ± 4.0	23.23 ± 4.3	*p* < 0.001^*∗*^
	Mean differences	8.8 ± 3.2	0.98 ± 6.1	*p* < 0.001^*∗*^
	Intragroup test results	*p* < 0.001^*∗∗*^	*p*=0.22^*∗∗*^	

^*∗*^Independent sample *t*-test; ^*∗∗*^pair sample *t*-test; *N*: frequency; *p*: *p* value; RLS: restless leg syndrome; SD: standard deviation.

**Table 3:** The mean score of fatigue before and after intervention in CCNs.

Variable		Intervention (*N* = 60)	Control (*N* = 60)	Intergroup test results
		Mean ± SD	Mean ± SD	
Fatigue	Preintervention	63.4 ± 6.8	63.4 ± 6.0	*P*=0.97^*∗*^
	Postintervention	59.6 ± 4.4	63.1 ± 5.6	*p* < 0.001^*∗*^
	Mean differences	4.1 ± 6.6	0.3 ± 7.8	*p* < 0.001^*∗*^
	Intragroup test results	*p* < 0.001^*∗∗*^	*P*=0.78^*∗∗*^	

^*∗*^Independent sample *t*-test; ^*∗∗*^pair sample *t*-test; *N*: frequency; *p*: *p* value; CCN: critical care nurse; SD: standard deviation.

## Data Availability

The datasets used and/or analyzed during the current study are available from the corresponding author upon reasonable request.
